# Mechanisms of Heat-Treatment-Induced Cracking in Additively Manufactured IN738 Alloy

**DOI:** 10.3390/ma16237316

**Published:** 2023-11-24

**Authors:** Kesong Miao, Ziyi Ding, Rengeng Li, Xia Ji, Xiutao Duan, Rui Yao, Peng Chen, Hao Wu

**Affiliations:** 1 Key-Laboratory for Light-weight Materials, Nanjing Tech University, Nanjing 211816, China; lirengeng@njtech.edu.cn (R.L.); 202161103008@njtech.edu.cn (R.Y.); chenpeng@njtech.edu.cn (P.C.); hwu@njtech.edu.cn (H.W.); 2 School of Materials Science and Engineering, Harbin Institute of Technology, Harbin 150001, China; dingziyi121@163.com; 3 Falcon Tech Co., Ltd., Wuxi 214000, China; jeexiastudy@163.com (X.J.); dxt98891@126.com (X.D.)

**Keywords:** laser powder bed fusion, Ni-based superalloys, cracking, heat treatment

## Abstract

The present study conducts a comprehensive study on heat-treatment-induced cracking of Inconel 738 (IN738) alloy fabricated by laser powder bed fusion (LPBF) using scanning electron microscopy (SEM), energy dispersion spectrum (EDS), and electron backscatter diffraction (EBSD). The results indicate that the macroscopic crack is dominantly triggered by the strain-age cracking mechanism and propagates along grain boundaries. The initiation of cracking is facilitated by the superimposition of residual stress induced by the LPBF process and contraction stress induced by precipitation, while the reopening of compress pores at grain boundaries weakens the grain boundaries and provides fast channels for cracking. These results revealed the coupling effects in triggering heat-treatment-induced cracking, offering a fundamental guideline for crack control during heat treatment of additively manufactured IN738 alloy.

## 1. Introduction

The IN738 alloy is widely used in aerospace gas turbine components in place of heat-resistant steels and IN718 [1], owing to its excellent properties, including high strength, heat resistance, and corrosion resistance [2,3]. The recent rapid development of metal additive manufacturing technologies has shed new light on the fabrication of IN738 components used in the aerospace industry due to its capacity to produce small batches efficiently and reduce machining steps [4,5]. Moreover, LPBF enables complex geometry of parts that traditional casts cannot reach, making it competitive against cast IN738 [6,7].

The primary additive manufacturing technologies adopted for IN738 include laser metal deposition (LMD), electron powder bed fusion (EPBF), and LPBF. The LMD is a process that involves producing melt tracks via relative movement between the deposition source and the workpiece [8]. Closed layers can be achieved by depositing multiple tracks next to each other and allowing suitable track overlap. The final parts with complex geometry were fabricated by depositing multiple layers on top of each other. The LMD IN738 typically exhibits directional epitaxial growth with cracks propagating along the build direction in grain boundaries alongside the presence of liquid film [9]. Although reducing laser power and scanning speed could be beneficial to reduce cracking in LMD [10,11], preheated substrates ranging from 700 to 1050 °C are required to achieve crack-free deposits [12]. The EPBF heats metal powders with a high-energy electron beam with preheated substrates, which melts and gradually builds into the desired geometry [13]. It is crucial to note that the process takes place under high vacuum and elevated bed-temperature conditions, typically around 1000 °C for Ni-based superalloys. It allows for low thermal gradients throughout the material, ensuring high-purity materials with minimal residual stress [14]. However, the parts fabricated with EPBF are limited in size and vacuum system maintenance requires additional expense, limiting its commercial application. This process of LPBF is analogous to that of EPBF, except that it utilizes a laser as the heating source, and generally, no preheating is employed [6]. The parts fabricated with LPBF show higher geometrical accuracy than those with LMD, meanwhile offering a wider range of size options compared to those with EPBF. The LPBF enables fast cooling rates, which generally result in finer grains and improved mechanical properties, accompanied by a higher level of residual stresses [15].

Although high-alloyed IN738 exhibits advanced mechanical properties, it also endures high crack sensitivity, which is further exacerbated by the high residual stresses and temperature gradients introduced by the LPBF process [16,17]. Most of the current research on LPBF IN738 has focused on the modification of scanning strategies to achieve crack-free components in an as-built state. For example, Cloot et al. investigated the crack formation of IN738LC processed by LPBF using Gaussian and doughnut profiles. It revealed that increasing scan speed could reduce cracking density at the expense of increasing porosity [18]. Wang et al. found that a medium energy input is suitable for achieving the best density [15]. In addition, they suggested that the scanning speed has a significant impact on the porosity, especially at the lower laser input. Zhou et al. reported that porosity minimization (<0.4%) is achieved when the energy densities range from 69 J/mm^3^ to 139 J/mm^3^ by evading the keyhole effect and lack of fusion [16]. During the rapid solidification of LPBF, precipitation-strengthening in Ni-based superalloys is inhibited due to the limited formation of the dominant strengthening phase, γ’ [19]. Thus, heat treatment for LPBF IN738 components is indispensable before application. However, the effect of postheat treatment is still an open question. Take hot isostatic pressing (HIP) as an example; some studies suggested that HIP could remove defects during the LBPF process and achieve better mechanical properties [20]. Other research reported that only internal defects can be closed while the surface defects are fully remained [21]. Furthermore, cracking after HIP treatment has also been reported [22], while heat-treatment-induced cracking is more common in other heat treatments [23,24]. However, less attention was paid to the mechanisms of heat-treatment-induced cracking in LPBF IN738.

The primary cracking modes in LPBF IN738 include solidification cracking, liquation cracking, ductility dip cracking, and strain-age cracking [25,26,27]. Solidification cracking generally occurs in the last period of solidification [18,28]. The high-temperature gradient induced by the LPBF process results in insufficient fluidity of the interdendritic liquid phase, which makes it challenging to coordinate the thermal shrinkage of the solid phase, thus inducing solidification cracking [29]. Liquation cracking occurs after the nominal solidification. Due to the rapid solidification process of LPBF, elemental segregation could present and form low melting point regions, leading to the appearance of liquid film when the bulk material is at an elevated temperature [27,30]. The above two mechanisms are accompanied by a liquid phase, which will leave characteristic features in the vicinity of the cracking. Ductility dip cracking is typically observed at temperatures within the range of 0.7 to 0.5 times the melting point of the alloy, where the alloy always suffers a rapid reduction in ductility [3]. The prevailing theories for ductility dip cracking involve grain boundary sliding and impurity embrittlement [10]. It is suggested that grain boundary morphology is vital to ductility dip cracking [10,31]. Long and straight grain boundaries exhibit low resistance to ductility dip cracking, which is caused by severe strain localization at triple grain boundary junctions [10]. In contrast, tortuous grain boundaries showed better susceptibility for ductility dip cracking [31]. Strain-age cracking is generally induced by the precipitation of hardening particles (e.g., γ’ phase for IN738 alloy) [10,27,31]. Volume shrinkage due to the precipitation will generate local stress at grain boundaries in polycrystalline, leading to higher susceptibility to intergranular cracking [27]. Moreover, sluggish precipitation kinetics is generally accepted to resist strain-age cracking, e.g., replacement of γ’ phase with slowly precipitated γ’’ phase as a strengthening source to achieve crack-free nickel-based alloys [3].

This study investigated the cracking behavior of the IN738 alloy that underwent heat treatment at 1130 °C for 3 h. A detailed microstructure characterization before and after heat treatment was systematically conducted, with extra care taken on the heat treatment induced cracking. Based on the results, mechanisms of heat treatment induced cracking in LPBF IN738 alloy and the effects of precipitate, pores, and residual stress on the cracking were discussed.

## 2. Materials and Methods

In this study, spherical IN738 powders with diameters of 15–45 μm were used as raw materials. The chemical composition of powders is shown in Table 1. The samples were prepared using the LPBF technique with EOS 290 equipment on a 316 L stainless steel substrate (chemical composition is listed in Table 2). The laser station used Yb-fiber laser as the source, providing a high power of up to 400 W and a focus diameter of 100 μm. The LPBF process parameters can be found in Table 3. These parameters achieved selective laser melting, i.e., the metal powders being liquefied [32]. After fabrication, the as-built sample (denoted as AB) was removed from the substrate and then subjected to heat treatment at 1130 °C for 3 h at a heating rate of 20 °C/min, followed by air cooling at a cooling rate of 50 °C/min (the sample denoted as HT). The heat treatment was carried out in a muffle furnace without inert gas, giving excellent resistance to oxidation of IN738 [33].

To observe the microstructure, the samples, with dimensions of 10 × 10 × 10 mm^3^, were subjected to mechanical grinding and polishing. The vertical planes of the AB and HT samples were ground with sandpaper ranging from 600 to 4000 grit and then polished to a smoothness of 0.05 μm on polishing clothes. The grain and precipitate characteristics were observed using a TESCAN scanning electron microscope (SEM), coupling with a backscattered electron (BSE) detector. The energy dispersive X-ray spectroscopy (EDS) analysis was performed with SEM equipment to analyze the chemical composition and distribution of elements. The electron backscatter diffraction (EBSD) measurements were performed with EDAX equipment mounted on the SEM equipment with a step size of 1 μm. The EBSD test results were processed and analyzed by Aztec software (Version 2.1). The specimens for transmission electron microscopy (TEM) analysis were prepared by the twin-jet electropolisher (TJ100-SE) with a solution of 10 vol.% perchloric acid and 90 vol.% ethanol at the temperature of −30 °C. The TEM characterization was carried out on JEOL JEM-F200 with a voltage of 200 kV. For the evaluation of mechanical properties, the Vickers hardness of the samples was measured using QNESS Q10 CHD Master+ with a load of 5 kg. The residual stresses in the samples were determined by X-ray diffraction using Proto LXRD, with the utilization of Mn Kα radiation operated at 30 kV and 25 mA.

## 3. Results

**Figure 1:**
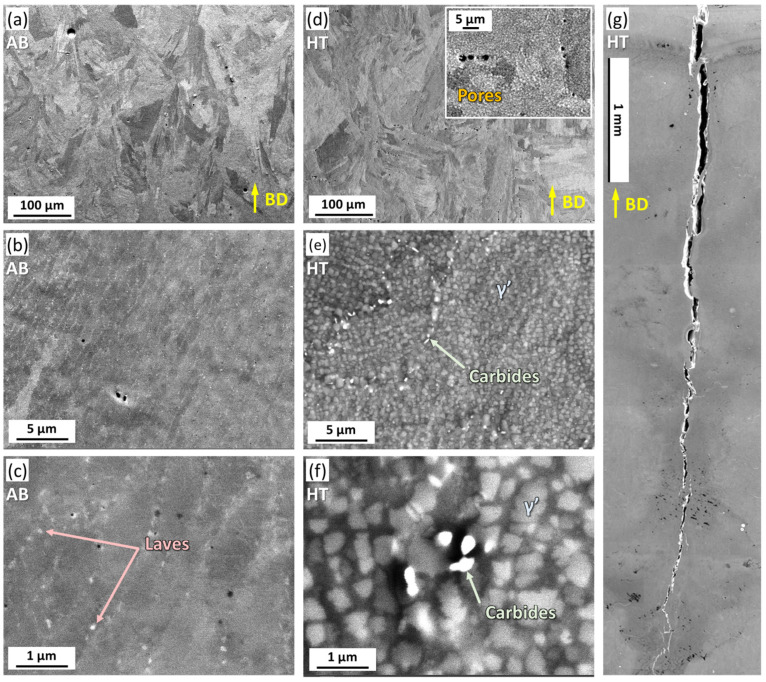
Microstructure of AB and HT samples. (**a**–**c**) BSE images of the AB sample with different magnifications. (**d**–**f**) BSE images of the HT sample with different magnifications. (**g**) SEM image of crack morphology in the HT sample.

**Figure 2:**
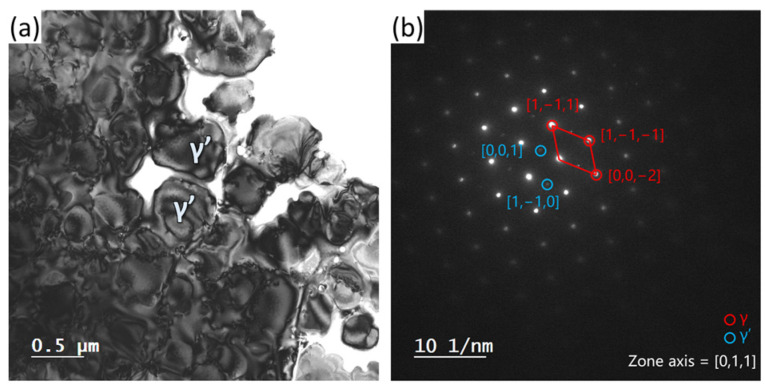
The TEM identification of precipitates. (**a**) Bright-field image. (**b**) Selected area electron diffraction.

The heat treatment for IN738 is generally designed for dissolving the Laves phase and generating precipitates. Figure 1 shows the microstructure of AB and HT samples, respectively. As shown in Figure 1a,b, the AB sample contained elongated cellular structures, which exhibited a preferred arrangement along the build direction (BD). The high-magnification image (Figure 1c) demonstrates the formation of granular precipitates in the cellular structure. The precipitates possessed typical structural characteristics of the Laves phase, which is generally reported in the literature [34]. After the heat treatment, the grain morphology slightly changed (Figure 1d), accompanied by the dissolution of the dendritic distribution of the Laves phase (Figure 1e). In addition, large particulate precipitates with a size of 200–860 nm, γ’, have uniformly precipitated within the grains. The formation of γ’ was further confirmed by TEM, as shown in Figure 2. The bright-field image (Figure 2a) demonstrates the size and morphology of γ’ that are consistent with that in the BSE images (Figure 1), while the γ’ phase was identified by selected area electron diffraction, as shown in Figure 2b.

**Figure 3:**
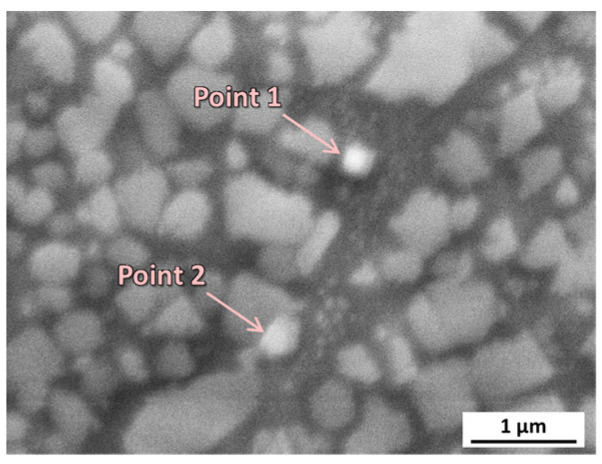
Schematic of EDS analysis for carbides.

The chemical compositions of AB and HT samples were further characterized by EDS and listed in Table 4. The B, C, and Zr were not included in the analysis due to the inaccuracy of EDS measurements for light and low-content elements. The compositions of AB and HT were consistent with that of the powder. It suggested that the constituent elements did not volatilize appreciably during the laser additive manufacturing process, owing to the high boiling points of the constituent elements. The main element composition of γ’ is Ni, Ti, and Al, showing a light-gray contrast in the BSE images (Figure 1e,f). Meanwhile, there were also small precipitates with a size of 170–280 nm (carbides) that precipitated at the grain boundaries. Due to the enrichment of elements such as Nb and Ta, the carbides show a bright contrast in Figure 1e,f, as marked by arrows. The EDS analysis was carried out on carbides (Figure 3), and the results are listed in Table 5. The enrichment of Ti, Nb, and Ta could be found, confirming they were MC-type carbides, which is consistent with other reports [35]. Due to the formation of precipitation and carbides, the hardness increased from 395.5 HV (AB sample) to 421.0 HV (HT sample). Figure 1g showed that macroscopic cracks with a length of more than 3 mm appeared at the top of the HT sample, initiated from the top surface and propagating along BD.

It is worth mentioning that compared to the AB sample, a number of spherical pores can be observed in the HT sample with random distribution with respect to BD, as shown in the inset of Figure 1d. To clarify that such bias is not induced due to the localized field of view, five rectangular areas with dimensions of 500 μm × 375 μm were randomly selected in the AB and HT samples, respectively, and the pore sizes in the areas were measured. Thus, the porosity of the samples can be obtained by dividing the pore area by the region area. The porosity of the AB sample is 0.5%, while as a comparison, the porosity of the HT sample increased to 1.3%. Both the spherical morphology and distribution of pores in the HT sample suggested that it might originate from the volume expansion of gas inside the sample under high-temperature conditions, and the formation process will be discussed in Section 4.2.

**Figure 4:**
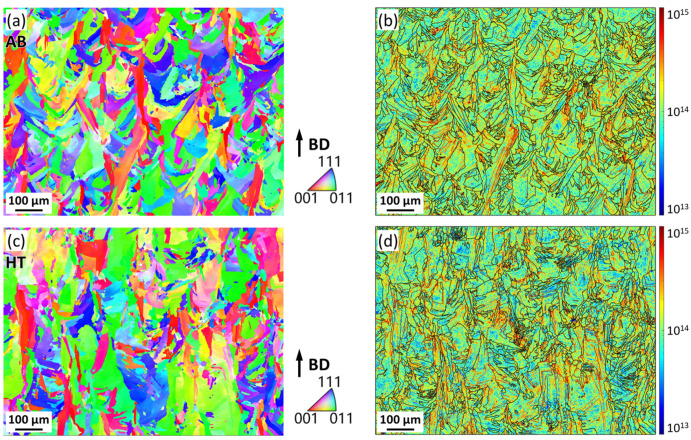
IPF maps of (**a**) AB sample and (**c**) HT sample; GND maps of (**b**) AB sample and (**d**) HT sample.

Figure 4a,c shows the inverse pole figure (IPF) maps of the AB sample and HT sample in the vertical direction, both featuring columnar crystals parallel to the BD. The AB sample exhibited an average grain size of 15.6 μm, while the HT sample exhibited an average grain size of 16.1 μm. It is indicated that there are no significant alterations in the grain size before and after the heat treatment, although the grain morphology slightly changed. Based on the EBSD data, the geometrically necessary dislocation (GND) maps were calculated according to the method proposed by W. Pantleon [36], and the results are shown in Figure 4b,d. The AB sample exhibited an average GND of 2.6 × 10^14^ mm^−2^, while the HT sample exhibited an average GND of 2.3 × 10^14^ mm^−2^, suggesting that the heat-treatment process does not significantly alter the GND density. Furthermore, the high dislocation density regions are mainly localized at grain boundaries in grains with relatively high length-to-width ratios and present a tendency to be parallel to the BD.

**Figure 5:**
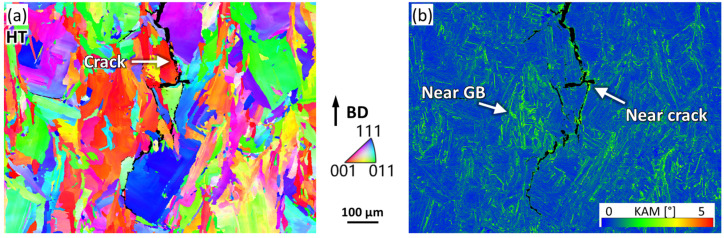
EBSD images of crack morphology in HT sample: (**a**) IPF map; (**b**) KAM map.

To clarify the mechanism of the heat-treatment-induced crack in Figure 1g, an EBSD scan was performed in the region near the crack tip, as shown in Figure 5a. The crack displayed a dominant tendency to propagate along the high-angle grain boundaries. Figure 5b shows the kernel average misorientation (KAM) map of the identical region, which is a measure of local plastic deformation. It is apparent that higher KAM is present on both sides of the crack, shown in green, in contrast to the low KAM region (shown in blue) in the grain interior. However, the deformation localization near the crack did not show distinct features compared with that near the high-angle grain boundaries, as highlighted by arrows in Figure 5b.

**Figure 6:**
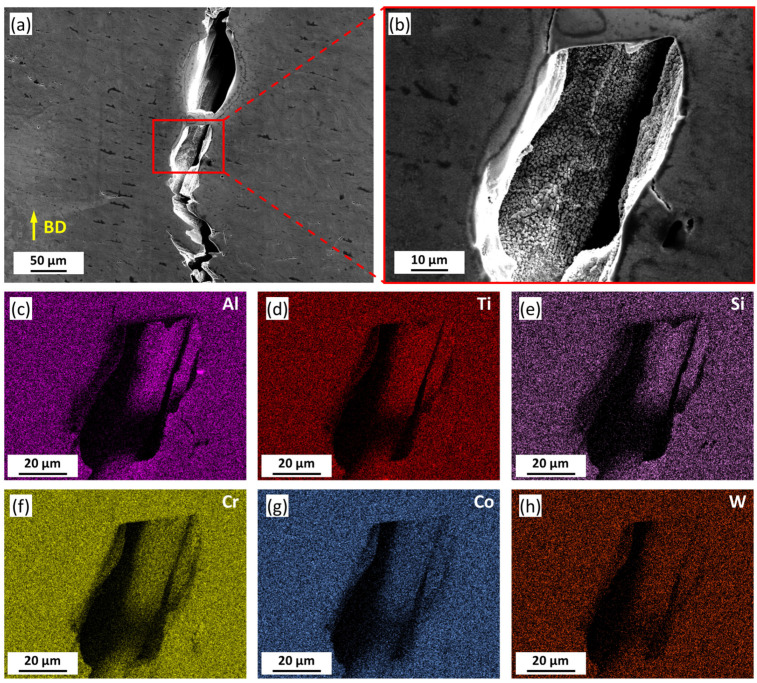
Microstructure of the crack surface in HT sample (**a**) low magnification SEM image; (**b**) high magnification SEM image. Element distribution of (**c**) Al, (**d**) Ti, (**e**) Si, (**f**) Cr, (**g**) Co, and (**h**) W.

Figure 6a shows the microstructure of the cracks in the HT sample, while the enlarged view of the crack surface is displayed in Figure 6b, as indicated by the red rectangle. The SEM images exhibited a high-density γ’ phase on the crack surface. It is worth noting that γ’ phases on the crack surface in the HT sample exhibited a larger size compared with that observed in Figure 1. Then, EDS mapping scans of the major alloying elements were executed in the identical area in Figure 6b. The distribution of Al, Ti, Si, Cr, Co, and W were characterized by EDS and are shown in Figure 6c–h, respectively. No significant elemental segregation was found near the crack. Although the EDS signals from some areas of crack surfaces were shaded due to geometrical relation, the collected information indicated the occurrence of Ti and Al enrichment on the crack surfaces.

**Figure 7:**
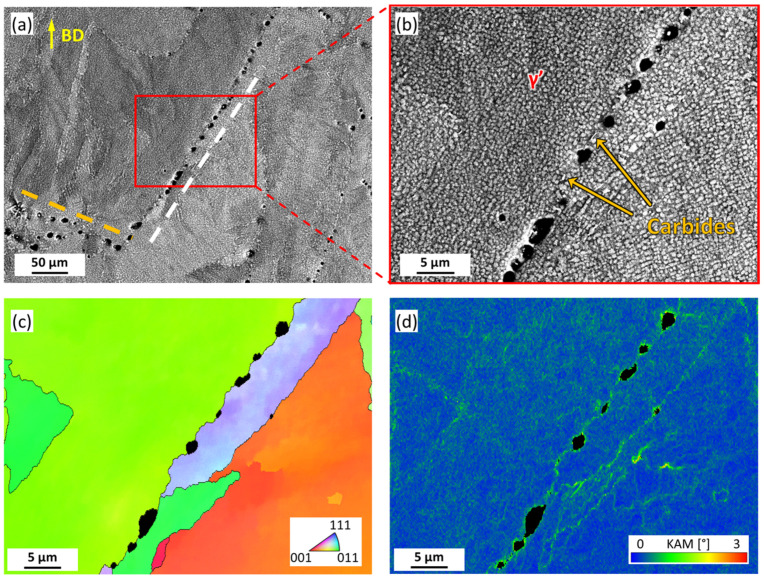
Intergranular microstructure of HT sample. (**a**) low magnification BSE image; (**b**) high magnification BSE image. (**c**) IPF map and (**d**) KAM map from EBSD results of identical region of (**b**).

Considering the features of crack propagation along the grain boundaries, the uncracked grain boundaries in the HT sample were further characterized, as shown in Figure 7a. It was found that the spherical pores observed in Figure 1d had a directional alignment feature. The alignment direction could be with a small angle to BD (as depicted by the white dashed line) or a large angle to BD (as depicted by the orange dashed line). Both types are not significantly different in terms of single pore morphology. Figure 7b presents a magnified image of the area highlighted by the red rectangle in Figure 7a. It was seen that the γ’ phase was uniformly distributed, independent of pores. Moreover, carbides with brighter contrast and different morphology can be observed between the pores, as emphasized by the arrows. The IPF map from EBSD results (Figure 7c) further identified that these pores are distributed at grain boundaries. It is worth mentioning that grain boundaries neighbored pores displayed higher curvature, i.e., form the more tortuous grain boundaries near pores. The KAM map of the identical regions suggests that deformation tends to accumulate at both grain boundaries and pore-grain interfaces, with the latter being more localized, as shown in Figure 7d.

## 4. Discussion

The AB sample was crack-free and had low porosity in the present study. After heat treatment at 1130 °C for 3 h, which is generally utilized as a solid solution process to dissolve Lave phases, macroscopic cracking occurred in the HT sample with the porosity increased simultaneously. The crack microstructure did not exhibit features of liquation or resolidification. Thus, solidification cracking and liquation cracking mechanisms can be ruled out. The results showed that cracking propagated along tortuous grain boundaries, accompanied by large-sized γ’ phase and elemental enrichment of Ti and Al on the crack surface. It is suggested that the current cracking could be attributed to strain-age cracking. The reason and the dominant factor that affected the cracking will be discussed in the following paragraphs.

### 4.1. The Effect of Precipitates

The γ’ phase plays a vital role in IN738 alloy as a primary strengthening source. The phase has a nominal chemical composition of Ni_3_(Al, Ti) and exhibits a low lattice parameter mismatch (coherent) with the nickel matrix, allowing fast precipitation kinetics. The effect of γ’ phase on crack susceptibility involves the following aspects: (i) Degradation of deformation capability. Despite the limited strengthening capacity reported for the large-sized γ’ phase [37], the precipitation of the γ’ phase led to the formation of dislocation loops by the Orowan mechanism, inducing weakening of the deformation capability [38,39]. Moreover, the precipitation reaction is suggested to contribute to the higher strength of the grain interior than the grain boundaries, facilitating the deformation (e.g., deformation induced by thermal stress) to be localized to the grain boundaries [27]. It is well supported by the GND distribution (Figure 4) and the KAM map (Figure 5 and Figure 7), where higher GND density and lattice misorientation near grain boundaries could be observed. (ii) Contraction stress due to γ’ phase precipitation. Rapid solidification for additive manufacturing resulted in the solid solution of Ti, Al, and other elements in the Ni matrix in IN738. As the heat treatment proceeded, the γ’ phase precipitated and consumed the Ti and Al in the Ni matrix. Since the atomic radius of Ti and Al is larger than that of Ni atoms, the process of Ni atoms refilling the positions taken by Ti and Al atoms causes the volume shrinkage of the Ni matrix. The intergranular mutual restraint in polycrystalline impeded the full release of volume shrinkage strain, inducing contraction stress on grain boundaries. It is noteworthy that the concentration of the elements forming the γ’ phase has an essential effect on cracking, which is well described by the Prager−Shira diagram [3]. The nickel-based alloys exhibited higher cracking susceptibility with increasing Ti and Al content. It has also been reported that higher Ti and Al content promote γ’ phase precipitation [2], leading to strain-age cracking by raising significant shrinkage stresses when the residual stresses are not sufficiently relieved. In addition, a few small-sized carbides were observed at grain boundaries. It is believed that they may serve as pinning for grain boundary sliding and control the grain size [40]. It could contribute to the formation of tortuous grain boundaries, as shown in Figure 7. It revealed that further compositional modulation or adjustment of the LPBF parameters to evade Ti and Al segregation is worth conducting in future work.

### 4.2. The Effect of Pores

On the one hand, pores at grain boundaries in the HT sample reduced the contact area between grains, lowered grain boundary strength, and provided fast channels for crack propagation, leading to the degradation of crack resistance. On the other hand, these pores facilitated the formation of tortuous grain boundaries. The tortuous grain boundaries could obstruct grain boundary sliding by interlocking, avoiding drastic strain localization at the triple junction of grain boundaries, as supported in Figure 7d. It was reported to be positive to evade ductility dip cracking [10,31]. However, the pore size in the present study is so large that the former detrimental mechanism took the primary role.

The mechanism for the increasing porosity of the HT sample is interpreted as follows: The laser additive manufacturing is processed in a flowing argon atmosphere. The melt pool undergoes directional motion driven by Marangoni convection, folding the liquid metal over the surface while simultaneously entraining argon bubbles [41]. During the solidification process, these pores may be compressed or even completely closed under the action of thermal shrinkage strain, leaving high internal gas pressure inside. With elevated temperatures, the pore pressure rose, and the strength of the Ni matrix dropped, facilitating pore growth [42]. Moreover, the contraction stress at grain boundaries, as discussed in Section 4.1, would also assist the pore growth. The spherical morphology is driven by reducing the stress concentration around pores. In addition, due to the obstacle effects of pores to dendrite growth in the melt, the dendrite growth direction is thus redirected, leading to a significant difference in orientation on both sides of the pores, which in turn resulted in the formation of grain boundaries along the pores [43], as shown in Figure 7. The current results suggest the need for precise regulation of LPBF parameters in future work to avoid the introduction of argon bubbles, even if they are extremely small in the as-built sample.

### 4.3. The Effect of Residual Stress

Both γ’ phase precipitation and pore formation are spatially randomly distributed, independent of BD (Figure 7), yet macroscopic cracks have an apparent tendency to propagate along the BD. It is suggested that additional factors contribute to the macroscopic cracking.

**Figure 8:**
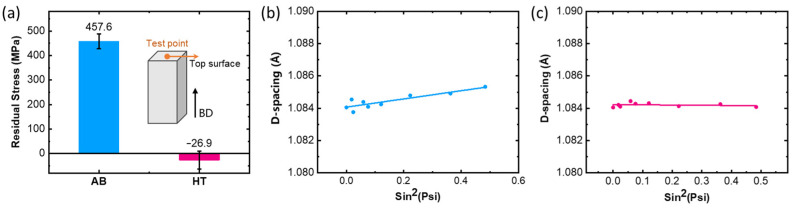
Residual stress in the AB and HT samples. (**a**) Schematic of the test point and the residual stress results. The d-spacing versus sin^2^(Psi) relationships of (**b**) AB sample and (**c**) HT sample.

Residual stress is acknowledged as a crucial factor promoting strain-age cracking, which is widely reported in welding [37,44,45,46]. During the cooling process following welding, thermal stresses and external weld restraints may cause notable residual stress to develop in the joint. In the case of the LPBF process, the rapid heating and cooling process can introduce high initial residual stresses into the material [47,48,49]. The residual stresses of the LPBF parts have proven to have spatial distribution patterns [50,51]. As the last area to solidify, the thermal shrinkage at the top area is limited by the presolidified layers [52,53,54,55,56], resulting in the tensile residual stress perpendicular to the BD. The residual stresses on the top surface of the AB and HT samples were measured via XRD. The schematic of the test point is illustrated as an insert in Figure 8a. It was suggested that tensile residual stresses of up to 457.6 MPa were present on the surface in the AB samples, which is perpendicular to the BD. After heat treatment of 1130 °C for 3 h, the complete relief of residual stress was achieved in the HT sample, consistent with the generally reported minimizing and homogenizing residual stress with heat treatment at temperatures above 1000 °C [57]. Such results were further confirmed by the d-spacing versus sin^2^(Psi) relationship shown in Figure 8b,c. It is also notable that although heat treatment can relieve residual stresses, the rapid precipitation of γ’ mentioned in Section 4.1 implies that contraction stress resulting from phase transformation will accumulate when the material remains at a high level of residual stress (not fully relieved by heat treatment). Whilst the grain boundary paralleled the BD, the residual stress direction was in line with the shrinkage stress direction, and the superimposed local stresses intensified, exceeding the grain boundary strength and inducing cracking. The remaining tensile residual stress in the early stages of heat treatment provided extra drive force for crack initiation and propagation along the BD. Future research on heat treatment optimization should consider taking low-temperature annealing as the first heat treatment to reduce residual stress while avoiding precipitation.

## 5. Conclusions

In this study, microstructure variations before and after heat treatment at 1130 °C for 3 h were systematically characterized to clarify the mechanism for heat-treatment-induced cracking in LPBF IN738. The following conclusions have been reached:

(i) The heat-treatment-induced cracking in the present study is attributed to strain-age cracking.
(ii) The crack propagated was driven by the superimposition of contraction stress induced by precipitation and residual stress induced by the LPBF process. The former is facilitated by local enrichment of Ti and Al in the early stage of heat treatment when the residual stress is not fully relieved by heat treatment.
(iii) The formation of large-sized pores after heat treatment is attributed to the reopening of compressed argon bubbles. The pores weaken the grain boundaries and provide fast channels for cracking.

To avoid heat-treatment-induced cracking, careful modifications are required for fabrication and heat-treatment processes to reduce residual stresses, and the sluggish kinetics of γ’ phase precipitation or eliminate pores.

## Figures and Tables

**Table 1:** Nominal chemical composition of IN738 powders.

Element	Ni	Al	Ti	Cr	Co	Mo	Nb	Ta	W	Zr	B	C
wt. %	Bal.	3.4	3.4	16.0	8.5	1.8	0.9	1.7	2.6	0.05	0.01	0.11

**Table 2:** Nominal chemical composition of 316 L stainless steel substrate.

Element	Fe	C	Mn	P	S	Si	Cr	Ni	Mo	N
wt. %	Bal.	0.08	2.00	0.045	0.03	0.75	16–18	10–14	2–3	0.1

**Table 3:** The LPBF process parameters.

Laser Power	Scan Speed	Layer Thickness	Hatch Length	Scan Strategy
280 W	980 mm/s	40 μm	100 μm	67° rotation

**Table 4:** Chemical compositions of AB and HT samples.

Sample	Element	Ni	Al	Ti	Cr	Co	Mo	Nb	Ta	W
AB	wt. %	Bal.	3.37	3.70	16.08	8.34	1.68	0.86	2.21	3.38
	at. %	Bal.	7.18	4.44	17.79	8.14	1.01	0.53	0.70	1.06
HT	wt. %	Bal.	3.37	3.70	16.06	8.37	1.67	0.83	2.26	3.26
	at. %	Bal.	7.17	4.44	17.77	8.16	1.00	0.52	0.72	1.03

**Table 5:** EDS analysis for carbides.

Position	Element	Ni	Al	Ti	Cr	Co	Mo	Nb	Ta	W
Point 1	wt. %	Bal.	1.02	10.87	16.33	7.24	0.80	3.33	8.92	2.16
Point 2	wt. %	Bal.	0.97	13.90	15.50	6.58	0.73	4.53	11.94	2.05

## Data Availability

The data presented in this study are available on request from the corresponding author.
